# Microneedle‐Assisted Delivery of PEGylated Recombinant Human Fusion Collagen for Androgenetic Alopecia: Preclinical and Pilot Clinical Evidence

**DOI:** 10.1111/jocd.70931

**Published:** 2026-06-04

**Authors:** Meitong Jin, Yanli Kong, Ling Ge, Yinli Luo, Chuying Li, Jingbi Meng, Zhehu Jin, Longquan Pi

**Affiliations:** ^1^ Department of Medical Cosmetology The Affiliated Hospital of Yanbian University (Yanbian Hospital) Yanji China; ^2^ Department of Dermatology The Affiliated Hospital of Yanbian University (Yanbian Hospital) Yanji China; ^3^ College of Medical Yanbian University Yanji China

**Keywords:** androgenetic alopecia, hair restoration, microneedling, PEGylated recombinant human fusion collagen (PEG‐rhCol‐F), Wnt/β‐catenin

## Abstract

**Background:**

Androgenetic alopecia (AGA), the most common form of progressive hair loss in men, significantly impairs both physical and psychological well‐being. While traditional research has predominantly focused on cellular components and signaling pathways, the extracellular matrix (ECM)—particularly collagen—is increasingly recognized as playing a dual role: serving as both a structural scaffold and a dynamic signaling reservoir for hair follicles. Consequently, a recombinant fusion collagen that integrates key functional domains of human types III and XVII collagen with hair follicle‐targeting sequences represents a promising strategy for ECM replenishment and functional restoration.

**Aims:**

This study aimed to evaluate the therapeutic feasibility and underlying mechanisms of microneedle‐assisted delivery of PEGylated recombinant human fusion collagen (PEG‐rhCol‐F) for AGA treatment.

**Methods:**

The therapeutic efficacy of microneedle‐delivered PEG‐rhCol‐F was first evaluated in a testosterone‐induced AGA mouse model through morphological, histological, and molecular analyzes (Western blotting). Subsequently, a three‐month pilot clinical trial involving 15 AGA patients was conducted. Patients received microneedling combined with a PEG‐rhCol‐F liquid dressing, and treatment outcomes were assessed via trichoscopy.

**Results:**

In the AGA mouse model, PEG‐rhCol‐F treatment significantly promoted hair regrowth, increased hair follicle count, and facilitated the transition to the anagen phase. Mechanistically, this was associated with the activation of the Wnt/β‐catenin‐VEGF axis. Correspondingly, the clinical trial demonstrated a marked improvement in hair density and diameter from baseline, along with a shift from vellus to terminal hairs. The treatment regimen was well‐tolerated, with only mild, transient adverse reactions (e.g., erythema) observed.

**Conclusions:**

This pilot study provides initial evidence for the efficacy and safety of microneedle‐assisted PEG‐rhCol‐F delivery for AGA patients. By combining structural ECM repair with functional pathway activation, this dual‐mechanism approach merits further clinical investigation.

## 1 Introduction

Androgenetic alopecia (AGA), the most common form of progressive hair loss in men, extends beyond cosmetic concerns to significantly impair psychosocial well‐being and quality of life [1, 2, 3]. The pathogenesis of AGA is characterized by androgen‐driven progressive miniaturization of genetically predisposed hair follicles, primarily mediated by dihydrotestosterone (DHT). This disruption alters the hair cycle by shortening the anagen (growth) phase and prolonging the telogen (resting) phase, resulting in follicular atrophy and the replacement of terminal hairs with vellus‐like strands [4, 5, 6]. Current mainstay therapies—oral finasteride and topical minoxidil—are limited by variable efficacy, requirement for lifelong administration, frequent relapse upon cessation, and potential side effects that compromise patient adherence [7]. Consequently, there is an urgent need for novel therapeutic strategies that directly target the structural and functional regeneration of hair follicles.

Emerging evidence indicates that extracellular matrix (ECM) components, particularly type III collagen, function as dynamic signaling reservoirs essential for hair follicle biology [8]. However, in AGA, metabolic dysregulation—especially involving type XVII collagen—compromises the follicular microenvironment and disrupts the integrity of the DP and stem cell niche. To address this, we employed a recombinant human fusion collagen that integrates key functional sequences from humanized types III and XVII collagen with hair follicle‐targeted motifs, designed to promote follicle regeneration and restore scalp homeostasis. Although recombinant collagen offers therapeutic potential, its clinical application is hindered by poor transdermal bioavailability. To overcome this barrier, we utilized polyethylene glycol (PEG) modification, which enhances collagen's biocompatibility and stability [9], and combined it with microneedling therapy. This physical enhancement method creates temporary microchannels in the stratum corneum, facilitating transdermal delivery while simultaneously stimulating endogenous collagen production in the dermis [10, 11].

Here, we systematically evaluated the therapeutic potential of microneedle‐assisted delivery of PEGylated recombinant human fusion collagen (PEG‐rhCol‐F). Using a testosterone‐induced AGA mouse model, we administered PEG‐rhCol‐F beginning on Day 1 post‐depilation and assessed therapeutic efficacy on Day 15 [12]. Based on promising preclinical data, we translated this strategy into a 3‐month pilot clinical trial involving 15 AGA patients. This early‐phase study provides initial evidence for the efficacy and safety of PEG‐rhCol‐F, supporting a novel dual‐mechanism strategy for AGA management.

## 2 Materials and Methods

### 2.1 Ethics Statement

This study was approved by the Medical Ethics Committee of the Affiliated Hospital of Yanbian University in accordance with the principles of the Declaration of Helsinki. Written informed consent was obtained from all participating patients prior to enrollment. All animal procedures were conducted in strict compliance with the guidelines of the Institutional Animal Care and Use Committee (IACUC) of Yanbian University and were approved by the committee.

### 2.2 Animal Experiments

A total of 50 six‐week‐old specific pathogen‐free (SPF) male C57BL/6J mice were obtained from the Laboratory Animal Center of Yanbian University (Jilin, China). Upon arrival, the mice weighed approximately 20 g. After a 1‐week acclimatization period under SPF conditions, the dorsal skin of each mouse was depilated on Day 7 to synchronize the hair cycle. All subsequent experimental procedures were performed under standardized SPF housing conditions.

#### 2.2.1 AGA Mouse Model Establishment and Validation

After anesthetization, depilatory cream was applied to the dorsal skin of the mice to create a depilated area measuring 2 × 3 cm. Ten mice were randomly divided into two groups (*n* = 5 per group): a Control group (daily topical application of PBS) and an AGA group (daily topical application of 0.5% testosterone solution) (Table 1) [13]. Dorsal skin photographs were acquired on days 0, 7, 10, 15, and 21 to document changes in skin color and hair regrowth. Hair growth was evaluated using a semi‐quantitative scoring system (0–4) based on the percentage of hair‐covered area and the length of regrown hair shafts: 0 = no growth (smooth, pink or black skin); 1 = slight growth (coverage < 25%); 2 = moderate growth (coverage 25%–50%); 3 = significant growth (coverage 50%–75%); 4 = full growth (coverage > 75%). Scoring was performed by two independent observers blinded to group allocation, and the mean score was recorded. Mice were euthanized on day 21, and skin tissue samples were harvested for hematoxylin and eosin (H&E) staining.

**TABLE 1:** Experimental protocol for AGA model establishment and sample sizes.

Group	Experimental mice, *n*	Intervention
Control	5	After depilating the skin on the backs of the mice, PBS solution was applied topically to the skin daily.
AGA	5	After depilating the skin on the backs of the mice, a 0.5% (w/v) testosterone solution was applied topically to the skin daily

#### 2.2.2 Therapeutic Intervention With Microneedling‐Assisted PEG‐rhCol‐F Delivery

Following the confirmation of successful AGA modeling, a separate cohort of mice was used to evaluate therapeutic efficacy. Mice were randomized into four groups (*n* = 5 per group): (1) AGA group (treated with PBS solution); (2) MN group (microneedling with PBS solution); (3) PEG group (microneedling with PEG‐rhCol‐F solution); and (4) Mino 2% group (treated with 2% minoxidil solution). Beginning on day 1 post‐depilation, treatments were administered every other day, concurrent with daily testosterone application for AGA maintenance. Microneedling was performed using a 0.5 mm roller, rolled once vertically and once horizontally over the depilated area (Table 2). Macroscopic photographs were acquired on days 0, 7, 10, and 15. Tissue collection and analytical procedures, including histological assessment and Western blot analysis for mechanistic evaluation, were performed on day 15.

**TABLE 2:** Experimental groups, interventions, and sample sizes for AGA treatment via microneedling‐assisted PEG‐rhCol‐F delivery.

Group	Experimental mice, *n*	Intervention
AGA	5	After depilating the skin on the backs of the mice, a 0.5% (w/v) testosterone solution was applied topically to the skin daily; every other day, a solution of PBS was applied evenly.
MN	5	After depilating the skin on the backs of the mice, a 0.5% (w/v) testosterone solution was applied topically to the skin daily; every other day, a solution of PBS was applied evenly, followed by rolling a 0.5 mm microneedle roller over the treated area once vertically (from top to bottom) and once horizontally (from left to right), after which the same solution was reapplied.
PEG	5	After depilating the skin on the backs of the mice, a 0.5% (w/v) testosterone solution was applied topically to the skin daily; every other day, a solution of PEGylated recombinant human fusion collagen was applied evenly, followed by rolling a 0.5 mm microneedle roller over the treated area once vertically (from top to bottom) and once horizontally (from left to right), after which the same solution was reapplied.
Mino	5	After depilating the skin on the backs of the mice, a 0.5% (w/v) testosterone solution was applied topically to the skin daily; every other day, a solution of 2% minoxidil tincture was applied evenly.

### 2.3 Patient Samples

A cohort of 15 patients diagnosed with AGA was recruited from the Department of Dermatology at Yanbian University Hospital. Inclusion criteria consisted of: (1) male or female patients aged between 18 and 60 years with a confirmed diagnosis of AGA; (2) a Hamilton–Norwood score of 2–4 for male participants; (3) a Ludwig score of 1–3 for female participants; and (4) provision of written informed consent (Table 3). Exclusion criteria included: (1) severe systemic or autoimmune disorders; (2) pregnancy or lactation; (3) a known history of keloid formation; and (4) any other condition considered inappropriate for participation by the investigators.

**TABLE 3:** Demographic and baseline characteristics.

Variable		Total
Age in years, mean ± SD (range)		34.27 ± 10.12 (23–52)
Gender	Male	6
	Female	9
Hamilton Norwood Scale	III	4
	IV	2
Ludwig Scale	I	3
	II	6

*Note:* Values in table are presented as the mean ± SD unless stated otherwise.

### 2.4 Clinical Treatment Protocol

Patients received treatment with a recombinant collagen liquid dressing (manufactured by Changchun Kedar Biotechnology Co. Ltd., China) for three months. The primary active component is PEGylated recombinant human fusion collagen; however, specific details regarding detailed physicochemical characterization data regarding the PEGylation process are proprietary to the manufacturer. According to the manufacturer's disclosure, the formulation consists of Carbomer, Triethanolamine, flavor‐masking agents, Disodium EDTA, and Purified Water as excipients, with PEG‐rhCol‐F serving as the active ingredient. These excipients are inert and not known to promote hair regrowth. Prior to each session, patients were instructed to wash their hair. The treatment protocol was as follows: Following standard disinfection, 2 mL of the liquid dressing was applied topically to the affected areas. Subsequently, an electric microneedling device (Dr. Pen, equipped with a 12‐needle cartridge) was used to perform microneedling to a depth of 0.5–1.0 mm, continuing until mild erythema or pinpoint bleeding was observed. An additional 3 mL of the dressing was then applied, and the scalp was massaged to ensure full absorption. Treatments were administered once every 2 weeks. Patients were instructed to avoid sun exposure and strenuous exercise for 24 h post‐treatment.

### 2.5 Efficacy Evaluation and Safety Monitoring

Macroscopic photographs were acquired using a Canon camera (Canon Inc., Japan), and trichoscopic images were obtained using a Dr. Camscope system (Dermat Company, China). Hair counts were performed at ×60 magnification, and mean hair diameter (mm) was measured at ×150 magnification. Photographic documentation and clinical assessments were conducted at each treatment session. At the final follow‐up visit, Visio Face images of the scalp were captured at sites corresponding to the baseline images. These images were independently evaluated by a physician blinded to the treatment protocol, who assigned a Physician Assessment Score (0 = poor, 1 = acceptable, 2 = good, 3 = excellent). To minimize evaluation bias, baseline and post‐treatment trichoscopic images were randomized and presented to the blinded evaluator in a non‐chronological order. Similarly, trichoscopic scans were performed in the same regions as the baseline assessment to quantify hair density and mean hair diameter. Patient satisfaction was documented using the Patient Assessment Score, which utilized the same grading scale (0–3). Throughout the study, patients were monitored for adverse events, including pain, erythema, hemorrhage, hyperpigmentation, and scarring. The severity and duration of any observed reactions were recorded.

### 2.6 Statistical Analysis

Statistical analysis and graphical representation were performed using GraphPad Prism 9.0 (GraphPad Software, San Diego, CA, USA). Continuous variables with a normal distribution are expressed as mean ± standard deviation (SD). Comparisons between two groups were performed using two‐tailed Student's *t*‐tests (unpaired *t*‐test for independent animal groups; paired *t*‐test for clinical data before and after treatment). Multiple group comparisons were analyzed by one‐way analysis of variance (ANOVA) followed by Tukey's post hoc test. Differences were considered statistically significant if *p <* 0.05 (*p < 0.05, **p < 0.01 and ***p < 0.001).

## 3 Results

### 3.1 Establishment and Validation of the AGA Mouse Model

**FIGURE 1:**
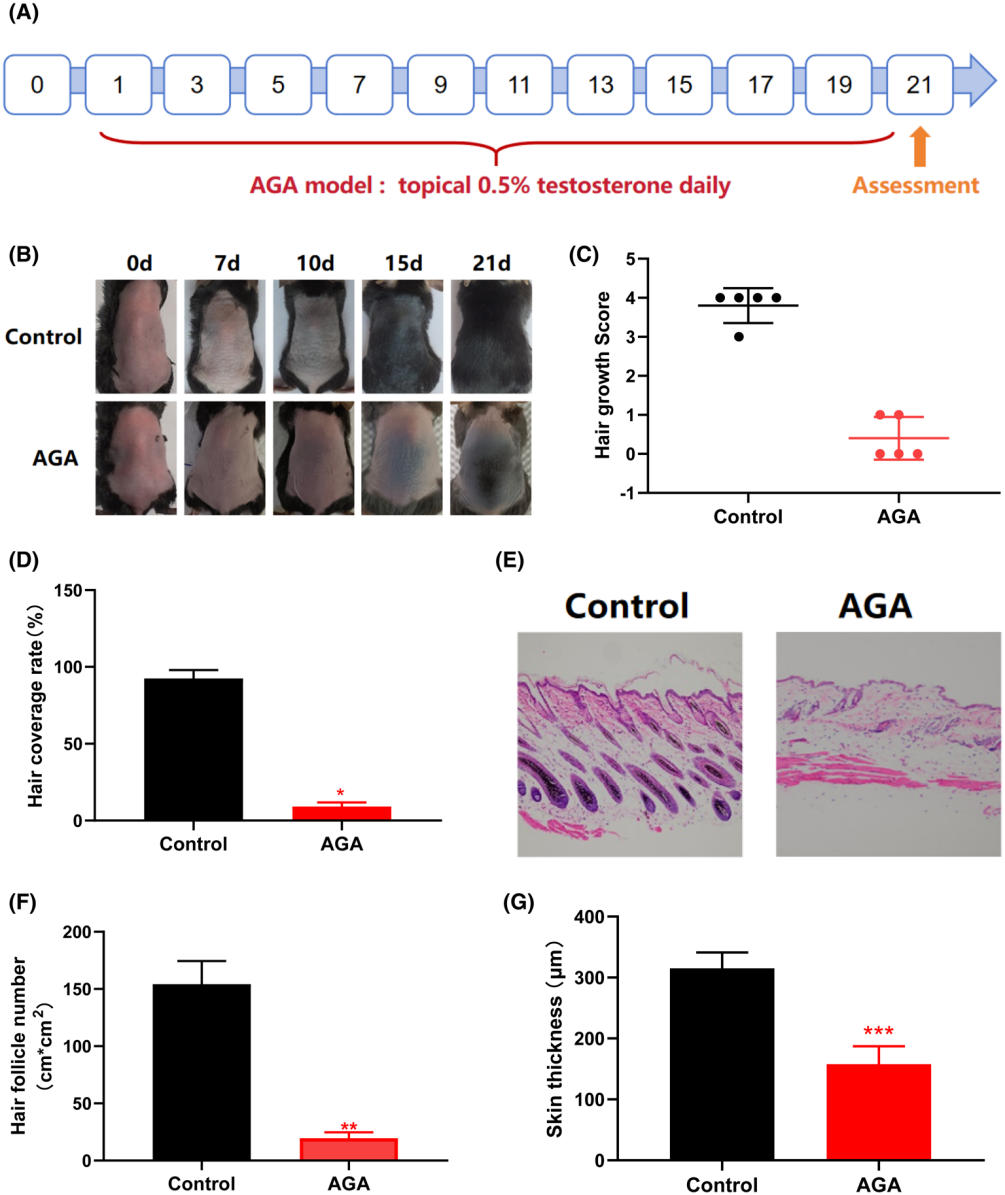
Establishment of AGA mouse model. (A) Timeline of AGA model establishment in C57BL/6 male mice. AGA was induced via daily topical application of 0.5% testosterone throughout the experiment. Hair regrowth was assessed on Day 21. (B) Representative optical images of testosterone‐treated mice. (C) Hair growth score of mice on day 21. (D) Hair coverage of mice on day 21. (E) H&E staining performed on day 21. Scale bars: 300 μm. (F) Quantification of hair follicle number (e.g., follicles/cm^2^) on day 21. (G) Quantification of skin thickness on day 21. Data are presented as mean ± SD (*n* = 5). Unpaired two‐tailed *t*‐test: *p < 0.05, **p < 0.01, ***p < 0.001 vs. control.

To evaluate the effects of PEG‐rhCol‐F on hair regrowth and explore the underlying mechanisms, a testosterone‐induced AGA mouse model was established (Figure 1A). As shown in Figure 1B, by Day 10 of induction, the AGA group exhibited no visible melanin pigmentation on the dorsal skin, whereas the control group displayed marked pigmentation. By Day 15, patchy hair regrowth was evident in the control group, while the AGA group showed only minor pigment aggregation. On Day 21, control mice achieved a hair growth score > 2 and hair coverage > 85%, whereas the AGA group had a score < 2 and significantly lower hair coverage compared with the control group (*p < 0.05; Figure 1C,D).

Histological analysis by H&E staining on Day 21 revealed that control mice had normal hair follicles with an even distribution and varying sizes, indicative of active proliferation and differentiation (Figures 1E). In contrast, the AGA group exhibited follicular atrophy and degeneration, with a significantly reduced follicle count (**p < 0.01; Figure 1F) and decreased skin thickness (***p < 0.001; Figure 1G) compared to controls. These results confirm the successful establishment and validation of the AGA mouse model.

### 3.2 Therapeutic Efficacy of PEG‐rhCol‐F Delivered via Microneedling in AGA Mice

**FIGURE 2:**
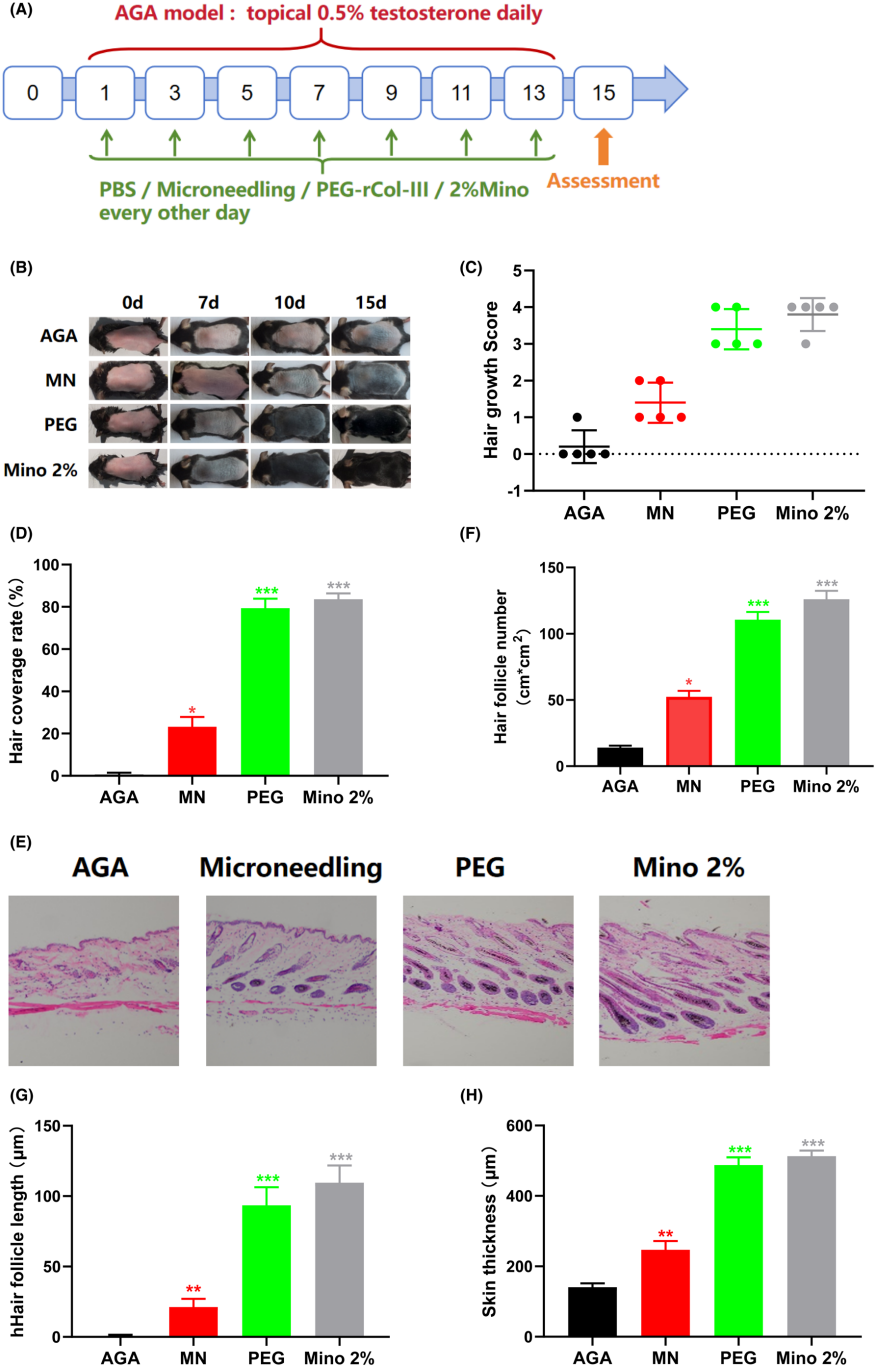
Evaluation of hair follicle regeneration in an AGA mouse model. (A) Schematic timeline of AGA model establishment and therapeutic intervention in mice. AGA was induced by daily topical application of 0.5% testosterone. Therapeutic interventions—treated with PBS (AGA group), Microneedling+PBS (MN group), Microneedling+PEG‐rhCol‐F (PEG group), and 2% Minoxidil (Mino group)—were administered every other day starting from Day 1, concurrently with model induction. Hair regrowth was assessed on Day 15. (B) Representative photographs of AGA mice treated with PBS (AGA group), Microneedling+PBS (MN group), Microneedling+PEG‐rhCol‐F (PEG group), and 2% Minoxidil (Mino group). (C) Hair growth score of mice on day 15. (D) Hair coverage rate of mice on day 15. (E) H&E staining performed on day 15. Scale bars: 300 μm. (F) Quantification of hair follicle number (e.g., follicles/cm^2^) on day 15. (G) Quantification of hair follicle length on day 15. (H) Quantification of skin thickness on day 15. Data are presented as mean ± SD (*n* = 5). One‐way ANOVA + Tukey's post hoc test: *p < 0.05, **p < 0.01, ***p < 0.001 vs. AGA group.

We next evaluated the therapeutic efficacy of PEG‐rhCol‐F administered via microneedling (Figure 2A). As shown in Figure 2B, by Day 10, both the PEG and Mino groups exhibited visible hair regrowth, whereas the AGA and MN groups displayed only localized skin pigmentation. By Day 15, the hair growth scores in the PEG and Mino groups exceeded 2 (Figure 2C), and hair coverage was > 75% in both groups—significantly higher than that in the AGA group (***p < 0.001; Figure 2D). Histological analysis by H&E staining revealed that, by day 15, the PEG group achieved dense hair regeneration (Figure 2E), with both hair follicle count and length significantly greater than those in the AGA group (***p < 0.001; Figure 2G,F). Regarding skin thickness, the AGA group showed the lowest values, while the MN group exhibited a modest but statistically significant increase (*p < 0.05; Figure 2H). In contrast, both the PEG and Mino groups demonstrated a significant increase in skin thickness compared to the AGA group (***p < 0.001; Figure 2H).

### 3.3 Modulation of the Wnt/β‐Catenin Signaling Pathway and VEGF Expression by PEG‐rhCol‐F

**FIGURE 3:**
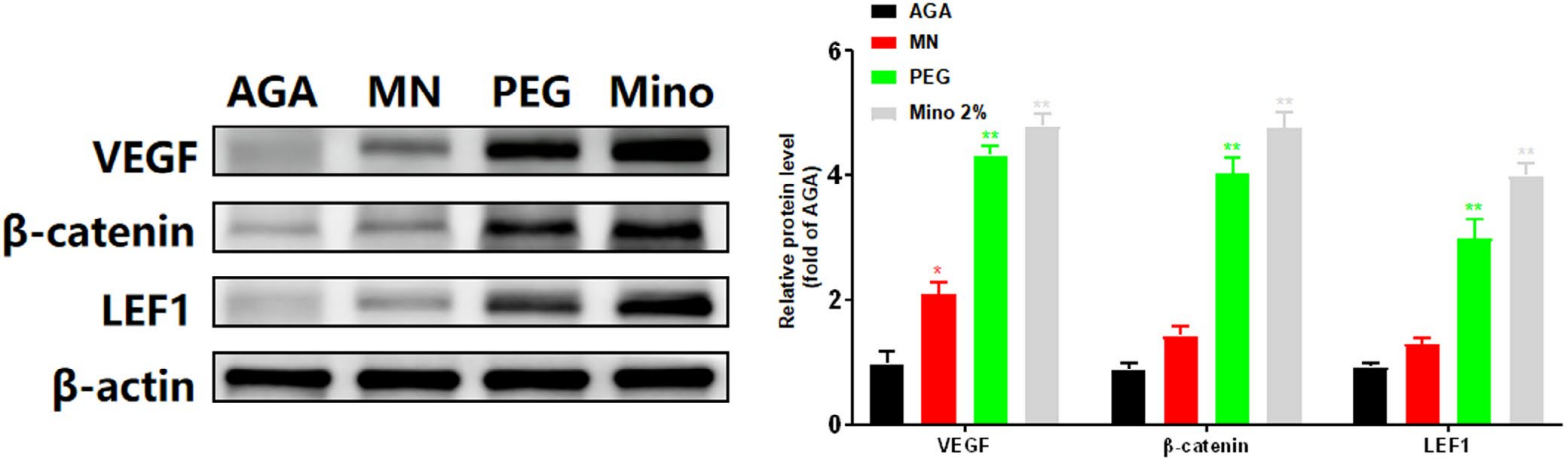
Modulation of the Wnt/β‐catenin signaling pathway and VEGF expression by PEG‐rhCol‐F. Protein expression and quantitation of VEGF, β‐Catenin, and LEF1 in each group on day 15. Data are presented as mean ± SD (*n* = 5), normalized to β‐actin and expressed as fold change vs. the AGA group. One‐way ANOVA + Tukey's post hoc test: *p < 0.05, **p < 0.01 vs. AGA group.

Western blot analysis of skin tissues harvested on day 15 revealed distinct molecular profiles among the treatment groups (Figure 3). Notably, the MN group exhibited a modest yet significant upregulation of VEGF compared to the AGA group (*p < 0.05), suggesting that microneedling alone induces a limited increase in VEGF protein levels. However, no significant differences were observed in the expression of β‐catenin or LEF1 in the MN group (*p* > 0.05), indicating that mechanical stimulation alone is insufficient to activate the canonical Wnt/β‐catenin signaling pathway. In contrast, the PEG and Mino groups exhibited a marked upregulation of VEGF, β‐catenin, and LEF1 compared to the AGA group (**p < 0.01; Figure 3). These findings suggest that while microneedling provides a modest upregulation of VEGF, PEG‐rhCol‐F serves as the primary driver of Wnt/β‐catenin pathway activation. Collectively, these results demonstrate that the combination of PEG‐rhCol‐F and microneedling elicits a superior therapeutic effect compared to microneedling alone.

### 3.4 Therapeutic Effect of Recombinant Collagen Liquid Dressing on Human Hair Growth

**FIGURE 4:**
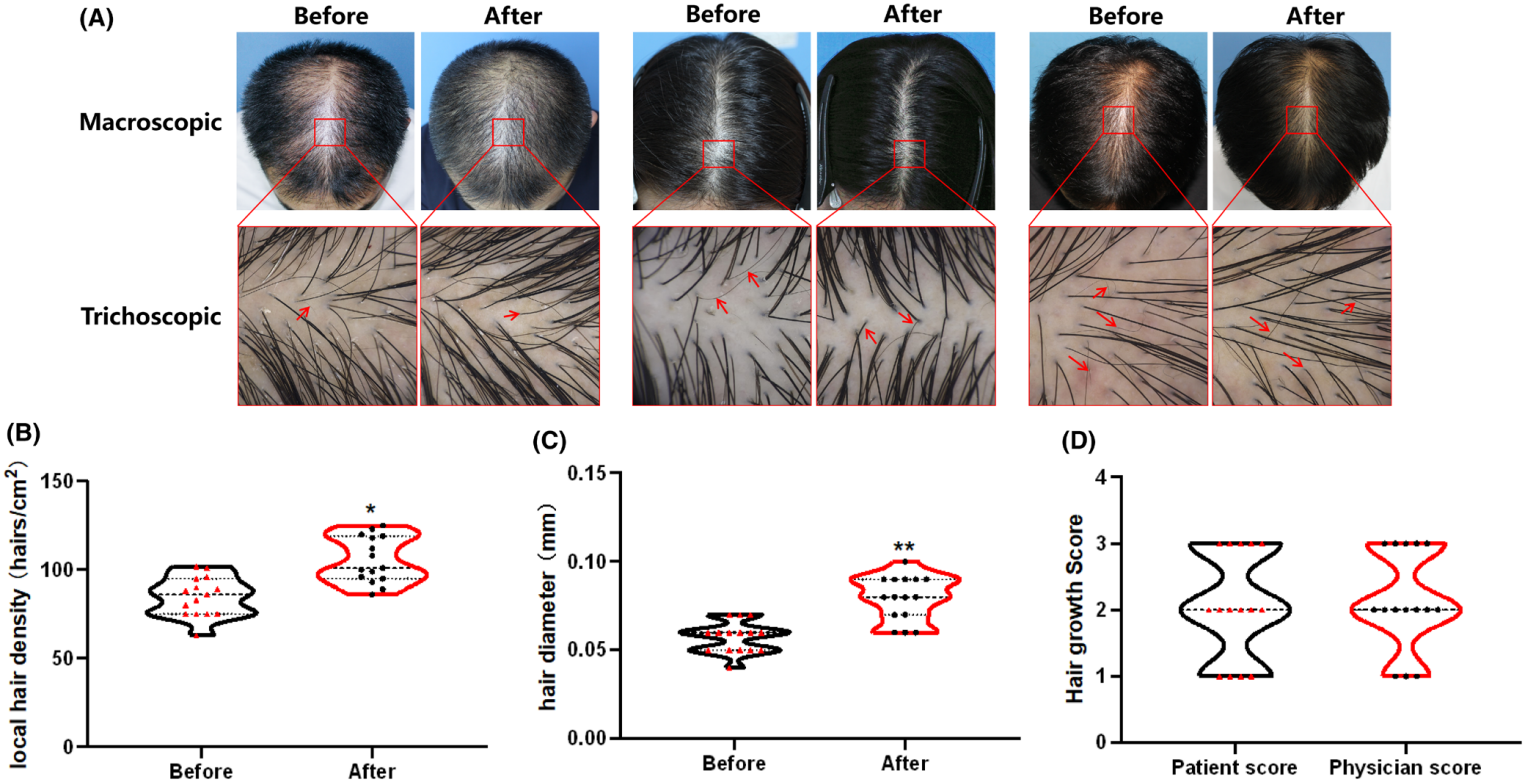
The effect of recombinant collagen liquid dressing on human hair growth. (A) Representative macroscopic views of the scalp (top row) and corresponding trichoscopic images (bottom row) from selected patients before and after treatment. Red boxes denote the regions of interest (ROIs) analyzed in the lower panels. Red arrowheads highlight the transition from vellus to terminal hairs. (B) Changes in hair density before and after treatment. (C) Changes in hair diameter before and after treatment. (D) Comparison of patient satisfaction scores and physician satisfaction scores. Data are presented as mean ± SD (*n* = 15). Paired *t*‐test: *p < 0.05; **p < 0.01 vs. before treatment.

By the end of the third month, all patients had completed the treatment and follow‐up protocol. No serious adverse events were reported during the study period. Macroscopic photographs demonstrated a visible increase in overall hair density compared with baseline (Figure 4A, top row). Paired trichoscopic images of the same regions of interest (ROIs, marked by red boxes) further elucidated these macroscopic changes. As shown in Figure 4A (bottom row), trichoscopic analysis revealed three key improvements: (1) a marked reduction in empty follicular openings, corresponding to increased hair density; (2) a clear shift from fine vellus hairs to thick, dark terminal hairs (highlighted by red arrowheads); and (3) a more uniform scalp background with reduced inflammation and keratinization.

Quantitative analysis using an intelligent scalp analysis system supported these visual findings. Mean hair density increased significantly from 84.87 ± 11.12 hairs/cm^2^ at baseline to 105.60 ± 13.05 hairs/cm^2^ (*p < 0.05; Figure 4B). Similarly, mean hair diameter increased significantly from 0.06 ± 0.01 to 0.08 ± 0.01 mm (**p < 0.01; Figure 4C). Regarding treatment outcomes, the mean patient‐reported assessment score was 2.20 ± 0.68, while the physician‐reported score was 2.33 ± 0.62. Both scores indicated moderate improvement, with no significant difference between the two assessment methods (*p* > 0.05; Figure 4D).

In terms of safety, patients experienced mild stinging during treatment. Two out of 15 patients (13.3%) developed transient erythema and pinpoint hemorrhages, which resolved spontaneously within 2 days. No hyperpigmentation or scarring was observed throughout the follow‐up period.

## 4 Discussion

This study provides the first systematic evaluation of the therapeutic potential of microneedle‐delivered PEG‐rhCol‐F in AGA. In a murine model, the treatment markedly increased hair density and synchronized the hair cycle toward the anagen phase. Mechanistically, exogenous collagen mediates this effect by directly replenishing and repairing the structural integrity of the dermal papilla and its ECM. This restoration establishes a supportive microenvironment for hair follicle stem cells, thereby laying the structural foundation for successful hair regeneration [14].

At the molecular level, we demonstrated that this combinatorial therapy significantly upregulates key signaling pathways. We observed a marked increase in β‐catenin and LEF1—core components of the Wnt/β‐catenin signaling pathway, which are indispensable for hair follicle development and stem cell maintenance [15, 16, 17, 18]. We speculate that exogenous recombinant human fusion collagen may function as a signaling molecule, triggering this cascade via integrin binding or alterations in the biomechanical properties of the microenvironment, thereby stabilizing this core effector [19, 20]. The coordinated upregulation of these proteins drives the transcription of downstream effectors, most notably VEGF [21, 22]. Consistent with this, the significant increase in VEGF protein expression suggests upregulated molecular signals and an improved follicular microenvironment. Collectively, these results demonstrate that PEG‐rhCol‐F is the primary driver of hair regrowth, orchestrating a dual mechanism involving both canonical Wnt/β‐catenin activation and robust VEGF‐mediated angiogenesis, while microneedling provides a permissive, pro‐regenerative microenvironment that enhances therapeutic delivery and efficacy.

Therefore, we propose a dual‐mechanism model for the observed hair‐promoting effect: while the collagen scaffold repairs the structural deficits of the follicular unit, it simultaneously reactivates the core signaling program that drives follicles into the growth phase. This mechanistic insight derived from animal models provides a plausible explanation for our clinical observations. In the 12‐week clinical trial, improvements in trichoscopic parameters (increased hair density, conversion of vellus to terminal hairs, and reduced scalp inflammation) and high patient satisfaction marked the successful translation of laboratory findings into clinical practice. The favorable tolerability and absence of significant adverse reactions further support the safety profile of this approach. This suggests that combining microneedling technology—a physical penetration‐enhancement method—with highly biocompatible recombinant collagen enables efficient, precise, and safe local delivery, thereby avoiding the potential risks associated with systemic administration.

However, this study has certain limitations. First, regarding the animal model, we utilized male C57BL/6 mice and testosterone—a well‐established method for inducing AGA in this strain [13, 15]. However, the short hair growth cycle in mice and the absence of humanized microenvironmental factors limit the full recapitulation of the complex pathophysiology of human AGA. Second, regarding mechanistic depth, while we identified the activation of the Wnt/β‐catenin pathway as the primary driver of hair regeneration mediated by PEG‐rhCol‐F, the precise molecular targets through which the fusion construct regulates this pathway remain to be fully characterized. Notably, while microneedling alone induced a modest increase in VEGF, PEG‐rhCol‐F treatment led to a more pronounced upregulation of both Wnt signaling and VEGF. Distinct from classical growth factors or single‐chain recombinant collagen, the PEG‐rhCol‐F construct likely exerts its effects through a dual mechanism: leveraging the specific bioactivity of its type XVII collagen domain while simultaneously modulating the biophysical properties of the ECM via the type III collagen backbone. Future investigations should extend beyond the Wnt pathway to explore potential crosstalk with other key regenerative pathways, such as the BMP and FGF signaling axes, to fully map the mechanistic landscape of collagen‐mediated hair follicle regeneration. Third, regarding the clinical trial, this was a pilot investigation with a relatively small sample size (*n* = 15) and a short observation period. Crucially, the absence of a clinical control group (either microneedling‐only or topical collagen‐only) limits our ability to definitively quantify the isolated contribution of PEG‐rhCol‐F versus the mechanical effects of microneedling. Although we prioritized patient safety by omitting a microneedling‐only arm to avoid unnecessary skin trauma, this limitation must be acknowledged. Larger‐scale RCTs with appropriate control arms are needed to confirm the specific efficacy of PEG‐rhCol‐F. Fourth, regarding product characterization, due to proprietary restrictions imposed by the manufacturer, detailed physicochemical characterization data regarding the PEGylation process could not be fully disclosed. This limitation may affect the reproducibility of the formulation by independent laboratories.

From a translational perspective, future research should prioritize the development of advanced delivery systems, such as dissolvable microneedle patches loaded with PEG‐rhCol‐F. Such platforms could achieve sustained drug release, ensuring optimal therapeutic concentrations from a single administration. This would significantly reduce treatment frequency, improve patient compliance, and minimize the risk of local adverse reactions—factors that are critical for the long‐term management of chronic conditions like AGA. Consequently, comprehensive long‐term safety evaluations will be a crucial next step to validate the clinical viability of this approach.

## 5 Conclusion

In summary, this pilot study provides initial evidence that microneedle‐assisted PEG‐rhCol‐F delivery represents a promising therapeutic strategy for AGA, operating through a dual mechanism of structural remodeling and signaling activation involving both Wnt/β‐catenin and VEGF pathways. This approach not only offers a potential new option for clinical hair loss treatment but also deepens our understanding of the multifaceted role of collagen in hair follicle biology, suggesting that it functions not merely as a static structural protein but also as a dynamic signaling regulator. Future investigations should focus on validating these findings in larger, controlled clinical trials and further elucidating the underlying molecular mechanisms.

## Conflicts of Interest

The authors declare no conflicts of interest.

## Data Availability

The data that support the findings of this study are available from the corresponding author upon reasonable request.
